# TRPV3 and Itch: The Role of TRPV3 in Chronic Pruritus according to Clinical and Experimental Evidence

**DOI:** 10.3390/ijms232314962

**Published:** 2022-11-29

**Authors:** Ji Young Um, Han Bi Kim, Jin Cheol Kim, Jin Seo Park, So Yeon Lee, Bo Young Chung, Chun Wook Park, Hye One Kim

**Affiliations:** Department of Dermatology, College of Medicine, Hallym University Kangnam Sacred Heart Hospital, Seoul 150-950, Republic of Korea

**Keywords:** transient receptor potential vanilloid-3, chronic pruritus, itching, atopic dermatitis, psoriasis

## Abstract

Itching is a sensory phenomenon characterized by an unpleasant sensation that makes you want to scratch the skin, and chronic itching diminishes the quality of life. In recent studies, multiple transient receptor potential (TRP) channels present in keratinocytes or nerve endings have been shown to engage in the propagation of itch signals in chronic dermatological or pruritic conditions, such as atopic dermatitis (AD) and psoriasis (PS). TRPV3, a member of the TRP family, is highly expressed in the epidermal keratinocytes. Normal TRPV3 signaling is essential for maintaining epidermal barrier homeostasis. In recent decades, many studies have suggested that TRPV3 contributes to detecting pruritus signals. Gain-of-function mutations in TRPV3 in mice and humans are characterized by severe itching, hyperkeratosis, and elevated total IgE levels. These studies suggest that TRPV3 is an important channel for skin itching. Preclinical studies have provided evidence to support the development of TRPV3 antagonists for treating inflammatory skin conditions, itchiness, and pain. This review explores the role of TRPV3 in chronic pruritus, collating clinical and experimental evidence. We also discuss underlying cellular and molecular mechanisms and explore the potential of TRPV3 antagonists as therapeutic agents.

## 1. Introduction

Itching, especially chronic pruritus, is a common symptom of dermatological and systemic diseases, and it severely affects the quality of life. Acute pruritus occurs on skin affected locally by insect bites and pruritus-causing substances, such as food, drugs, and physical allergens, whereas chronic pruritus occurs in several skin diseases, such as atopic dermatitis (AD), systemic conditions, and infectious diseases and they are also affected by immune diseases [1]. AD is a chronic inflammatory skin disease that requires long-term treatment [2]. It induces scratching, exacerbating skin inflammation, and causes pain [3,4], which in turn irritates the skin and causes itching. In addition, a large majority of patients suffer from itching and accompanying symptoms, including sleep disturbance and reduced quality of life; these physical symptoms are often accompanied by psychological symptoms such as stress and depression due to apparent skin lesions [5].

Post-burn pruritus, a typical result of scarring in burn patients, is one type of chronic pruritus [6]. After a burn, pruritus develops as the burn wound is being treated and healed. Different neuropeptides that are released by the skin’s nerves during wound healing regulate the growth of connective tissue cells, vascular cells, and epidermal cells. A range of inflammatory substances are secreted by activated keratinocytes in response to an itch-inducing substance, making them more vulnerable to itch receptors.

Various transient receptor potential (TRP) ion channels are involved in the molecular mechanisms of itching [7]. TRP ion channels play an important role in the propagation of itch signals in chronic dermatological conditions, such as AD and psoriasis (PS) [4]. Activation of the transient receptor potential vanilloid-3 (TRPV3) in skin keratinocytes regulates diverse cutaneous functions, including skin barrier formation [8], hair growth [9], wound healing [10], temperature sensing [11], and itch and pain perception [12]. The crucial significance of this channel in skin health and disease has been made clear by gain-of-function genetic mutations in TRPV3 in rats and humans [13,14,15]. TRP ion channels participate in several sensory processes, including pain [12]. TRPV3 is expressed in various tissues and organs, but the most prominent expression of TRPV3 occurs in epithelial cells of the skin [3,4]. TRPV3 also has a temperature-sensing function; it is activated at lower temperatures (33–39 °C) than TRPV1, and it shows a pronounced response to harmful temperatures. This makes TRPV3 sensitive to thermal stimulation or repeated application of activators [15].

The identification of selective TRPV3 activators and inhibitors may potentially lead to beneficial pharmacological interventions for pruritus. Numerous drugs, including antihistamines, local anesthetics, and opioid antagonists, have been used to improve itching. However, currently available antipruritic agents have limited selectivity and potential side effects, and their clinical effects have been mostly unsatisfactory [6].

Therefore, it is necessary to review the overall progress in the field and conduct further research on TRPV3–itch linkage. The purpose of this review is to explore the role of TRPV3 in chronic pruritic dermatitis based on clinical and experimental evidence and to discuss its cellular and molecular mechanisms. This review will assist in drug development and help improve the treatment of patients.

## 2. Gene and Structure of TRPV3 Channels

The vanilloid TRP family comprises TRPV1–TRPV6 and constitutes a subgroup of the TRP superfamily. TRP ion channels were first discovered in trp-mutant strains of *Drosophila* [16], which have been shown to exhibit transient potential elevations in response to light stimuli; therefore, they are termed transient receptor potential channels. TRPV3 is a human gene with 18 exons that is found near TRPV1 on chromosome 17p13. Similar to human TRPV3, mouse TRPV3 is found on chromosome 11B4 and has 18 exons [17]. The DNA sequence of the mouse or human TRPV3 gene’s entire open reading frame, according to Smith et al., is 2373 bp long and encodes a polypeptide with 791 amino acids. However, it is known that TRPV3 is selectively spliced in humans to produce variants with 790, 791, and 765 amino acids [17].

The TRP channels also vary considerably in their structures. However, certain shared domains can also be grouped together. The TRP channel consists of four subunits, each containing six transmembrane segments (S1–S6) with intracellular N- and C-terminus [18]. The hydrophilic loop between S5 and S6 forms an ionically conductive pore. Numerous post-transcriptional mechanisms, including phosphorylation, G-protein receptor coupling, ligand gating, and ubiquitination, are used to activate and regulate TRP channels in response to a variety of stimuli [19]. The majority of these receptors, which are found in virtually all cell types, are situated on the membranes of cells and organelles to modulate ion influx [20]. In addition, the amino acids located before the pores confer channel selectivity. This channel is non-selective for cations but prefers calcium; TRP channels have negatively charged residues in their pores that attract positively charged ions [20].

TRPV3 has cytoplasmic amino and carboxyl termini, ankyrin repeats, coiled-coil domains at the amino terminus, six trans-membrane segments, a reentrant pore loop, and numerous potential phosphorylation sites [21]. Compared to the ankyrin repeat domains of TRPV1 and TRPV4, TRPV3 is thought to have six ankyrin repeats, an insertion in repeat 1, and two brief deletions in repeats 4 and 5 [22]. TRPV1 is an ion channel expressed in sensory neurons that induces cation influx, and TRPV4 is an ion channel that is broadly expressed and activated by chemical, osmotic, and mechanical stimuli. It is known to regulate cellular signals related to various physiological and pathological processes by regulating Ca^2+^ entry. All six TRPV subfamily members have an ankyrin repeat domain (ARD) in their intracellular N-termini. The 33 residues that make up each ankyrin repeat often serve as motifs important for subunit–subunit interactions. Hydrogen bonding and hydrophobic packing sustain the distinctive bent finger 3 loop of TRPV3, and it is known that the overall structure of the TRPV3-ARD is comparable to that of the ARDs of other members of the TRPV subfamily [21].

### 2.1. Functions of TRPV3 Channels

Most TRP ion channels form homo- or hetero-tetramers when completely functional [19]. They are known to regulate the driving force for ion entry and the Ca^2+^ and Mg^2+^ transport machinery [23]. TRP channels also function as intracellular calcium release channels, and thus play an important role in organelle regulation. TRP channels are known to interact with other proteins and play an important role in organelle regulation by forming signaling complexes, often of unknown pathways [23]. Most TRP channels play an important role in mediating various sensations, such as pain, temperature, various types of taste, pressure, and vision [21]. In the body, some TRP channels function as microscopic thermometers, detecting heat or cold [21]. TRP channels also sense osmotic pressure, volume, stretching, and vibration [24]. They have been identified as having complex, multidimensional roles in sensory signaling.

Among multidimensional roles, the thermo-TRP channel has a C-terminal domain responsible for thermo-sensation and a specific interchangeable region capable of sensing temperature stimuli to the ligand regulation process [25]. There are at least six different types of thermo-TRP channels, each with a different role. TRPM8 is implicated in cold-sensing mechanisms, TRPV1 and TRPM3 contribute to heat and inflammatory sensations, and TRPA1 promotes many signaling pathways, such as those involved in sensory transmission, nociception, inflammation, and oxidative stress [25].

Ferrer-Montiel A et al. reported that TRPV3 showed a distinct activation temperature at >33 °C; in contrast, TRPV1, TRPV2, and TRPV4 (i.e., other temperature-sensitive TRPV channels) were activated at >43 °C, >52 °C, and >30 °C, respectively [26]. However, TRPV3, as a thermal sensor, is poorly detected in the dorsal root ganglion (DRG) or trigeminal ganglion (TG) sensory neurons in rodents [27]. According to this report, keratinocytes release diffusive molecules, suggesting that TRPV3 can activate free nerve endings in adjacent DRG neurons when activated at temperatures >32 °C in the skin [28]. Mandadi et al. obtained TRPV1-deficient DRG neurons by incubating them with keratinocytes [28]. Only when keratinocytes were co-cultured with DRG sensory neurons did the cytoplasmic concentration of Ca^2+^ increase with heating, and suramin P2 purinergic antagonists and pyridoxalphosphate-6-azophenyl-2′,4′-disulfonic acid tetrasodium salt were able to prevent this rise in intracellular calcium.

### 2.2. Expression of TRPV3 Channels

TRP channels are a group of ion channels located primarily in the plasma membranes of many animal cell types [29]. In particular, TRPV3 was most abundantly expressed in keratinocytes of the skin and in the oral and nasal epithelium of both humans and mice, as indicated by in situ hybridization and reverse transcription polymerase chain reaction analysis [30,31,32].

TRPV3 plays an important role in forming the skin barrier by regulating the activity of transglutaminase, a Ca^2+^-dependent cross-linking enzyme group essential for keratinization [8]. The functional role of TRPV3 ion channels in the regulation of human hair growth was confirmed by Borbíró et al. In this study, we confirmed that TRPV3 activation inhibits human hair growth [9]. Aijima et al. identified TRPV3 as an essential receptor for heat-induced oral epithelial proliferation and wound healing [10]. 

As hypertrophic scarring is also associated with pruritus, we investigated the mechanism of skin fibrosis prevention. The results showed that the activation of TRPV3 channels regulates dermal fibrosis by reducing extracellular matrix production via the TRPV3-TSLP (Thymic stromal lymphopoietin) and TSLP-Smad2/3 pathways in dermal fibroblasts [30]. This mechanism was confirmed by treatment with carvacrol, a TRPV3 agonist. The regulation of Ca^2+^ influx and the expression levels of NFAT (nuclear factor of activated T-cells) and p-Smad2/3 were significantly elevated in carvacrol-treated dermal fibroblasts. ECM (extracellular matrix) production is induced via the TRPV3-TSLP-Smad2/3 pathway. Therefore, the TRPV3-TSLP-Smad2/3 pathways in dermal fibroblasts modulate dermal fibrosis by decreasing the synthesis of ECM [30].

In addition, TRPV3 was expressed not only in skin cells but also in other cells (human brain, spinal cord, DRG, TG, and testes); however, Peier et al. confirmed that it was not highly expressed in rodent DRG or TG [31]. TRPV3 is highly expressed in infiltrating eosinophils and the mucosal epithelium of nasal polyps [32]. TRP channels are highly expressed in cancer cells [33]. TRPV3 is present in the mitochondrial region [16]. Another study elucidated the endogenous presence of TRPV3 channels in the sperm of all species [34]. TRPV subfamily members were found to be endogenously expressed in the human T cell line Jurkat, primary human T cells, and primary murine splenic T cells by Majhi et al. [34]. In addition, Yan et al. confirmed that TRPV3 is expressed in human cirrhosis tissues [34]. TRP ion channels also participate in pain and temperature sensations [21]. Several studies have confirmed a role for TRPV3 in pain modulation. McGaraughty et al. reported that structurally diverse TRPV3 receptor antagonists reduced the response of spinal wide dynamic range (WDR) neurons to low-intensity mechanical stimulation in mice with neuropathic pain, but only central nervous system (CNS)-penetrating antagonists reduced spontaneous firing. TRPV3 receptor antagonism in general and especially inhibits the activity of key classes of neurons within pain pathways in a way consistent with the attempt to limit pathological nociceptive signaling, and is controlled by receptors in the periphery and brain. As a result, blocking TRPV3 receptors is likely to be an effective treatment for mechanical allodynia and pain [25].

TRPV3 activity in the brain has also been identified, and identified features include influencing food intake, maintaining neuronal homeostatic thermostats, and inducing memory, learning, and depression [35]. Several of these functions could have been related to the pain response. Butler and Finn firmly established an association between anxiety levels and pain states. Fujioka et al. and Trübel et al. reported that changes in the regional brain temperature may also alter somatosensory thresholds.

### 2.3. Genetic Studies of TRPV3 Channels

Genetic studies of TRPV3 channels include Olmsted syndrome (OS), a rare hyperkeratotic skin channelopathy of TRPV3 gain-of-function changes. It is a rare disorder characterized by a combination of periorificial and keratotic plaques and bilateral palmoplantar keratoderma. Mutation of TRPV3 is most often limited to the endoplasmic reticulum (ER) [36]. This also severely damages vesicle trafficking, causing reduced surface expression of these gene mutations and other cellular proteins [37]. TRPV3 antagonists may be useful because TRPV3 overactivity in OS may potentially cause skin inflammatory reactions and skin diseases. The normal development of skin barrier and immune regulation was found to be poor in a TRPV3-deficient mouse model, however, indicating a possible role for TRPV3 antagonists.

Yoshioka et al. reported that the Gly573Ser substitution in TRPV3 leads to allergic and pruritic dermatitis. This suggests that TRPV3 (Gly573Ser) plays a potential role in itching and scratching-related dermatitis. According to a study by Fatima et al., scratching events in response to various pruritogens were dramatically decreased in Trpv3 ^G573S^ mice compared to wild-type littermates [37]. Another experiment was conducted using ICR^TRPV3-/-^ mice to determine the maximal therapeutic effect of TRPV3 on specific types of itching [38].

## 3. Mechanisms of TRPV3 Channel Activation in Pruritus

Animal studies suggest that TRP ion channels play an important role in itch transmission. In a study by Qi, H. et al., it was found that mice used in an AD model, as well as skin biopsies from individuals with atopic dermatitis, had higher TRPV3 and PAR2 levels. In a mouse AD model, it has been reported that these genes’ modulation decreased scratching and inflammatory responses [38]. In a study by Qi, H. et al., TRPV3 channel inhibitors were identified as potential treatments for dermatitis and chronic pruritus [39,40,41]. Carvacrol, a natural TRPV3 activator, has been reported to cause pruritus in mice [42]. Therefore, we investigated the increase in itching caused by TRPV3 activators [38,42]. After carvacrol, a TRPV3 activator, was applied to burn scars, the correlation between the expression of TRPV3 activator and itching was evaluated [41,42]. This study confirmed that carvacrol can induce itching when applied to burn scars. Furthermore, carvacrol-induced itching was relieved 20 min after the application of forsythia extract and olopatadine solution and 24 h after application of tacrolimus ointment and *Scutellaria baicalenis* extract [43]. Sherkheli et al. reported that monoterpenoids cause agonist-specific desensitization of TRPV3 [44]. Cassandra et al. have suggested that drofenine is a novel TRPV3 agonist. Drofenine showed efficacy comparable to the known TRPV3 agonists 2-aminoethoxydiphenylboronate (2-APB) and carvacrol in HEK-293 cells [45]. There are also reports of the activation of TRPV3 by wood smoke particles [46]. Ethyl vanillin (EVA) and farnesyl pyrophosphate (FPP) are TRPV3 agonists [47].

### 3.1. TRPV3 Channels and Atopic Dermatitis (AD)

A study related to TRPV3 in itching caused by AD has been conducted [4]. The potential role of TRPV3 in pruritus has been previously elucidated in humans and mice in several studies. Seo et al. reported that TRPV3 is upregulated in the skin of an MC903-induced AD mouse model. They also confirmed that thermal stimulation induced enhanced secretion of thymic stromal lymphopoietin (TSLP), nerve growth factor (NGF), and prostaglandin E2 (PGE2) by keratinocytes in patients with AD through TRPV3 activation. These results suggest that TRPV3 is a potential therapeutic target for heat-induced itch in AD [4]. In addition, Qu, Y. et al. reported that inhibition of the TRPV3 channel attenuates AD [40]. They suggested that warm temperature-activated Ca^2+^ permeable TRPV3 channels engage in the pathogenesis of AD and that TRPV3 protein, along with the inflammatory factors tumor necrosis factor (TNF)-α and interleukin (IL)-6, were involved. They found that the TRPV3 channel is upregulated in mouse models of AD. Similarly, Larkin et al. reported a clear relationship between TRPV3 and AD [2]. Additionally, TSLP, IL-31, IL-22, IL-17, and IL-22 all contribute to the pathogenesis of AD. By transcriptional and channel modulation, IL-31 sensitizes TRPV1 and indirectly controls TRPV3 in keratinocytes [48]. The transcription of TRPV3 in keratinocytes can be upregulated and its activity can be enhanced by IL-31-induced BNP release from sensory neurons [48]. Another study investigated the effect of TRPV3 as a dendritic cell modulator on the pathogenesis of AD [2]. According to a study by Vasas, N. et al., TRPV3 is a pro-inflammatory ion channel, and its overexpression was demonstrated to significantly increase TRPV3-specific ions in cultured non-lesional AD keratinocytes in contrast to normal cells [49]. Imura, K. et al. also investigated the role of TRPV3 in the immune response during the development of dermatitis [50]. These results suggested that TRPV3 may be a therapeutic target for pruritic dermatitis. In a study by Yamamoto-Kasai et al., TRPV3 could play an important role in skin itching in patients with pruritus and dry skin in AD [38]. Spontaneous scratching was induced by acetone–ether–water (AEW) application and locomotor activity in ICR^TRPV3+/+^ and ICR^TRPV3−/−^ mice. It was exciting that our research has found tacrolimus ointment to have an inhibitory effect on pruritus brought on by carvacrol [40,43]. Tacrolimus, an AD treatment option, is known to bind FK-binding protein in calcium-calmodulin signaling and block dephosphorylation of NFAT [51]. Carvacrol’s inhibition of dephosphorylation reduces the itching it causes [30]. The inhibition of TSLP transcription may be the cause of the *Scutellaria baicalensis* extract’s inhibitory action on pruritus.

### 3.2. TRPV3 Channel and Psoriasis (PS)

Ozcan, S.S. et al. reported that TRP channels expressed in immune cells have been shown to be associated with inflammatory diseases [52]. *TRPM4, TRPM7, TRPV3, TRPV4*, and *TRPC6* mRNA expression levels in peripheral blood mononuclear cells (PBMCs) of patients with PS were investigated. Different expression patterns of TRP channels may play a role in the pathogenesis of PS. Therefore, further studies are required.

### 3.3. TRPV3 Channels and Post-Burn Pruritus

We found that itching after burns is a common cause of scarring in burn patients, and about 87% of burn patients complain of itching. Carvacrol is crucial for controlling cell migration [53]. These findings indicate to a potential pathogenic mechanism wherein over regulation of the TRPV3 channel during wound healing might result in hypertrophic scarring [53]. Our results confirmed that TRPV3 is mainly expressed in the epidermis of pruritic burn scar tissues [41]. In addition, an increase in intracellular Ca^2+^ levels, induction of TRPV3 activation, and an increase in TSLP expression level were confirmed in each sample collected from burn patients; this was not observed in normal tissues [6]. We also identified the TRPV3-Itch mechanism, in which the itch-related factor PAR2 was reduced by antagonism of TRPV3. Inhibition of protein kinase A (PKA) and protein kinase C (PKC) reduced TRPV3 function. In addition, it was confirmed that TSLP mRNA, protein, and TSLPR protein expression levels were elevated in post-burn pruritic scars compared to normal tissues [6,54].

### 3.4. Activation of TRPV3 Channels

TRPV3 is activated by warm temperatures, synthetic small-molecule chemicals, and natural compounds from plants. In particular, various natural compounds such as carvacrol, thymol, and eugenol directly activate TRPV3 channels [55]. Several other monoterpenoids that cause warmth or skin sensitization can also open channels [56]. Interestingly, 2-Aminoethoxydiphenyl borate (2-APB) is a mixed activator and inhibitor of TRPV3, acting as an inhibitor at low concentrations, but as an activator at higher concentrations [57]. Drofenine, a 2-APB analog, also acts as a TRPV3 selective agonist [45]. It is assumed to increase the levels of protein TRPV3.

## 4. Therapeutic Potential of Synthetic TRPV3 Inhibitors

As for the clinical trials of TRPV3 antagonists known to date, GRC15300 entered phase 2 clinical trials in 2012 as a treatment for neuropathic pain by Broad, L.M. [58] (Table 1). However, these trials were suspended by the end of 2013. No further developments were reported. Additionally, in the preclinical development stage, Glenmark discovered that GRC 15133 was the first compound to provide evidence of anti-hyperalgesic activity owing to TRPV3 blockade in an animal pain model, and GRC 17173 was the first oral medication to show potent and selective oral efficacy in all pain models tested. In preclinical studies, these antagonists have generated sufficient evidence to justify clinical studies to investigate the potential of TRPV3 blockers [58]. Hydra Biosciences Inc. has issued several patents (compound #64 and #82) for TRPV3 antagonists [58]. Among them, FTP-THQ, a potent and selective TRPV3 receptor antagonist, was evaluated in several in vitro and in vivo assays [58]. In addition, Neuberger et al. found that dyclonine hydrochloride improved inflammation, abrasions, and broken lesions of mucous membranes and skin, as well as relieved pain and itching in patients [59]. TRPV3 can be potently and selectively inhibited by dyclonine, an aromatic ketone that is FDA-approved as a clinical anesthetic and antipruritic for topical application [60]. In two distinct nerve pain models and a reserpine model of central pain, the selective TRPV3 antagonist 74a had an advantageous preclinical profile [60]. Icilin functions as a TRPV3 antagonist, as well as a TRPM8 agonist, and can modulate pain with dual actions [61]. Trpvicin, a synthetic TRPV3 antagonist, works by inhibiting TRPV3 channels by stabilizing them in the closed state and has been shown to have pharmacological potential to reduce pruritus in mouse models [62]. We believe that TRPV3 antagonists may be effective therapeutic agents, and further studies should be conducted for drug discovery and development in the future.

### Natural TRPV3 Inhibitors Have Therapeutic Potential

Kang et al. confirmed the inhibitory effect of *Tribulus terrestris* fruit extract on AD [63] (Table 2). The mechanism of action was found to be related to the regulation of calcium channels and the activation of mast cells. Nam et al. revealed that *Spirodela polyrhiza* (SH) extract modulates the activation of the AD-associated TRPV3 ion channel and inhibits mast cell degranulation [64]. They evaluated the effect of the SH extract in a HEK293T cell line overexpressing TRPV3. SH extract may treat abnormal skin barrier pathologies in AD by modulating the activities of the Orai1 and TRPV3 calcium ion channels and inhibiting mast cell degranulation. Recently, Qi et al. demonstrated the attenuation of AD by two natural isochlorogenic acid isomers [39]. In a previous study, Zhang et al. found that forsythoside B, an active phenylethanoid glycoside from *Forsythia suspense*, attenuated pruritus and keratinocyte death in mice through the selective inhibition of TRPV3 channels [65]. We also investigated the itch-suppression effect of *Forsythia* while studying a TRPV3 agonist [64]. Another study investigated the pruritus and anti-inflammatory effects of a natural verbascoside through temperature-sensitive Ca^2+^-permeable TRPV3 channels [66]. In addition, the efficacy of scutellarein (Scu) for dermatitis was evaluated in a mouse model of 2,4-dinitrofluorobenzene (DNFB)- and carvacrol-induced dermatitis [67]. These data demonstrate that Scu inhibits carvacrol-induced cell proliferation, expression, and release of chemotactic factors, and subsequently chemotaxis of PBMCs on HaCaT keratinocytes. A unique natural inhibitory compound targeted at TRPV3 is called the epimer 17R-RvD1. By injecting 17R-RvD1 locally, acute pain behaviors unique to TRPV3 were reduced [68]. Metabolites of omega-3 fatty acids, eicosapentaenoic acid (for RvE1), and docosahexaenoic acid (for RvD2) (for RvD2 and 17R-RvD1) include Resolvin E1 (RvE1), RvD2, and 17R-RvD1 [68,69]. Citrusinine-II, a naturally occurring acrid alkaloid from *Atalantia monophylla*, has been demonstrated by Han et al. to be a powerful and specific antagonist of the TRPV3 channel, which is essential to the pathophysiology of pruritus [70]. Both in vivo and in vitro, citrusinine-II demonstrated excellent anti-pruritic efficacy with low adverse effects. These findings point to the potential of a therapeutic that targets citrusinine-II to treat itch, pain, and other skin problems [70].

## 5. Conclusions

A recent in vitro and in vivo animal study evaluated the association of TRPV3 with pruritus in inflammatory skin diseases, such as atopic dermatitis and psoriasis. Nevertheless, little is known about the clinical relevance of TRPV3 and the pruritus-related pathway in humans. Therefore, further studies on the physiological and pathological actions and roles of TRPV3 in the human skin are required. Discovering therapeutic agents that modulate and inhibit TRPV3 may be the key to ameliorating or overcoming chronic pruritus.

## Figures and Tables

**Table 1:** Synthetic TRPV3 inhibitors.

Synthetic Compound (Ref.)	Status	Treated
GRC15300 [58]	Phase II (Discontinued)	Neuropathic pain, skin disorders
GRC 15133 [58]	Pre-clinical	Hyperalgesic activity
GRC 17173 [58]	Pre-clinical	All pain
Compound #64 and #82 [58]	Hydra patents	Thermal hyperalgesia
FTP-THQ [58]	Pre-clinical animal models	Scratching and pain behavior
Dyclonine hydrochloride [59,60]	FDA-approved	Anesthetic and antipruritic
74a [60]	Proven pre-clinical profile	Neuropathic pain, central pain
Icilin [61]	Not clinically validated	Pain
Trpvicin [62]	Not clinically validated	Itch, hair loss

**Table 2:** Natural TRPV3 inhibitors.

Natual Compound (Ref.)	Source	Treated
Isochlorogenic acid isomers [39]	*Forsythia suspensa*	AD
Tribulus terrestris [63]	fruit extract, small leafy plant	AD, the regulation of calcium channels and TRPV3
Spirodelae Herba (SH) [64]	*Spirodela polyrhiza*	AD, modulation of TRPV3, skin barrier pathologies in AD
Forsythoside B [65]	*Lamiophlomis rotate* or *Forsythia suspensa*	Pruritus, selective inhibition of TRPV3 channels
Verbascoside [66]	*Lamiales*	Pruritus and anti-inflammatory effects
Scutellarein [67]	*Scutellaria lateriflora, genus Scutellaria, fern Asplenium belangeri*	Dermatitis, skin inflammation
17R-RvD1 [68,69]	mammalian tissues	Acute pain behaviours
Citrusinine-II [70]	*Atalantia monophylla*	Pruritus, pain and other skin complaints

## Data Availability

Not applicable.

## Abbreviations

TRPV3	Transient receptor potential vanilloid-3;
AD	Atopic dermatitis;
PS	Psoriasis;
ARD	An ankyrin repeat domain;
PBMC	peripheral blood mononuclear cell;
TSLP	Thymic stromal lymphopoietin;
NGF	nerve growth factor;
PGE2	prostaglandin E2;
PKA	protein kinase A;
PKC	protein kinase C;
TNF	tumor necrosis factor NFAT, nuclear factor of activated T-cells;
2-APB	2-Aminoethoxydiphenyl borate.
